# Supplementary material for: A generalizable cross-continent prediction of esophageal squamous cell carcinoma using the oral microbiome
Source: bioRxiv. 2025 Nov 24:2025.11.23.690048. Preprint. [Version 1] doi: 10.1101/2025.11.23.690048 (PMC12694582; doi:10.1101/2025.11.23.690048)
Supplement: 1 [file NIHPP2025.11.23.690048V1-supplement-1.pdf]

bioRxiv preprint doi: https://doi.org/10.1101/2025.11.23.690048; this version posted November 24, 2025. The copyright holder for this preprint (which was not certified by peer review) is the author/funder, who has granted bioRxiv a license to display the preprint in perpetuity. It is made available under aCC-BY 4.0 International license.

Supplementary figures

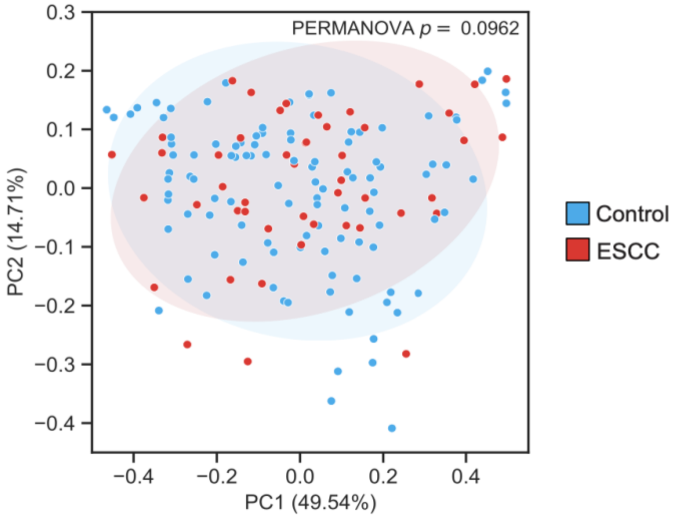

Supplementary Figure 1 | Weighted UniFrac PCoA demonstrated no association with ESCC.

PCoA on Weighted UniFrac distances demonstrated no significant clustering of patients with ESCC (PERMANOVA P = 0.096). Ellipses represent 2 standard deviations.

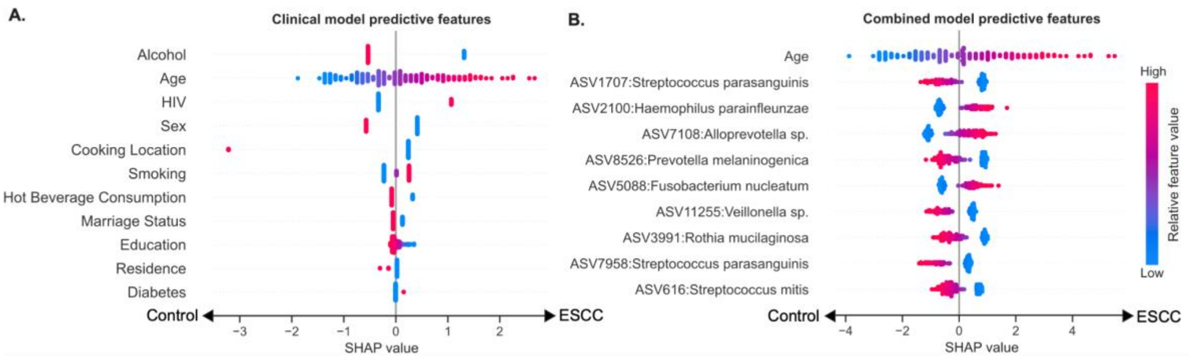

Supplementary Figure 2 | Top predictive features in the clinical and combined model

Effect on prediction (SHAP values) for the topmost predictive covariates in the **A.** clinical model and the **B.** combined clinical and microbiome-based model, with features sorted by importance. Each dot in the plots represents a specific sample, with the color corresponding to the value of the feature in the sample compared to all other samples.

bioRxiv preprint doi: https://doi.org/10.1101/2025.11.23.690048; this version posted November 24, 2025. The copyright holder for this preprint (which was not certified by peer review) is the author/funder, who has granted bioRxiv a license to display the preprint in perpetuity. It is made available under aCC-BY 4.0 International license.

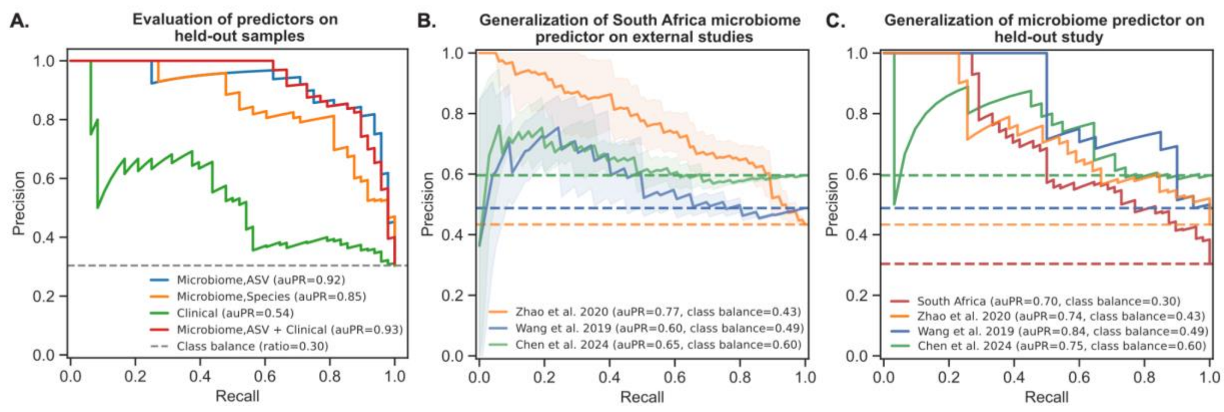

Supplementary Figure 3 | Microbiome-based classification of ESCC

A. Precision-recall (PR) curves showing the classification accuracy for ASV-level microbiome-based models (auPR=0.92),species-level models (auPR=0.85), models based on clinical data (auPR=0.54), and models based on both microbiome (ASV) and clinical data (auPR=0.93), all evaluated on held-out experimental processing batches. **B.** PR curves showing the classification accuracy of species-level microbiome predictors of ESCC, trained on our cohort (N=158) and evaluated separately on held-out studies from China: Zhao et al. 2020 (N=91), Wang et al. 2019 (N=41), and Chen et al. 2024 (N = 52). The model from each external cross-validation fold was evaluated separately, with the line showing the mean PR curve and shaded regions representing ±1 standard deviation. **C.** PR curves showing the performance of a species-level microbiome-based ESCC classifier, evaluated on each held-out study. The class balance for each study is shown as a dashed horizontal line.

bioRxiv preprint doi: https://doi.org/10.1101/2025.11.23.690048; this version posted November 24, 2025. The copyright holder for this preprint (which was not certified by peer review) is the author/funder, who has granted bioRxiv a license to display the preprint in perpetuity. It is made available under aCC-BY 4.0 International license.

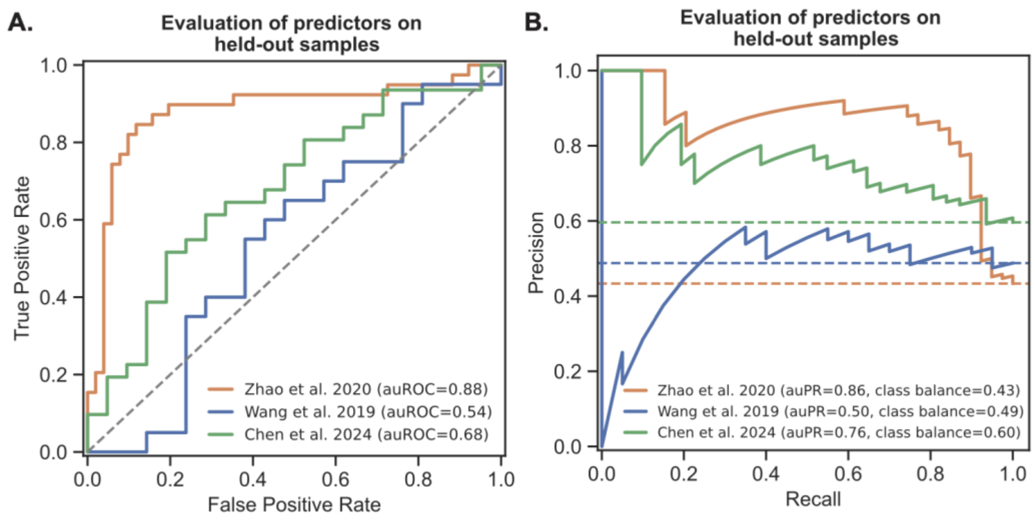

Supplementary Figure 4 | Microbiome-based classification of ESCC in external studies
A,B. Receiver operating characteristic (ROC) curves (A) and precision-recall (PR) curves (B) showing the classification accuracy of species-level microbiome-based models, evaluated on held-out samples using 10-fold nested cross-validation within each external study for Zhao et al. 2020, Wang et al. 2019, and Chen et al. 2024.

bioRxiv preprint doi: https://doi.org/10.1101/2025.11.23.690048; this version posted November 24, 2025. The copyright holder for this preprint (which was not certified by peer review) is the author/funder, who has granted bioRxiv a license to display the preprint in perpetuity. It is made available under aCC-BY 4.0 International license.

Supplementary tables

Supplementary Table 1 Patient characteristics

	ESCC (N=48)	Control (N=110)	P-value[1]
**Age (years;** mean (SD)**)**	59 (± 10)		<0.001
**Sex**			0.67
Male	26 (54%)	63 (57%)	
Female	20 (42%)	45 (41%)	
Missing	2 (4%)	2 (2%)	
**Marriage Status**			0.30
Married	19 (40%)	32 (29%)	
Not Married	29 (60%)	76 (69%)	
Missing	0 (0%)	2 (2%)	
**Location of residence**			0.36
Urban	40 (83%)	100 (91%)	
Rural	5 (11%)	4 (3%)	
Peri-urban	1 (2%)	3 (3%)	
Missing	2 (4%)	3 (3%)	
**Cooking location**			0.030
Inside	45 (94%)	97 (88%)	
Outside	0 (0%)	11 (10%)	
Missing	3 (6%)	2 (2%)	
**Highest level of education completed**			0.014
None	2 (4%)	2 (2%)	
Primary school	7 (15%)	7 (6%)	
Secondary school	31 (65%)	96 (87%)	
Higher Education	5 (10%)	3 (3%)	
Missing	2 (6%)	2 (2%)	
**History of smoking tobacco**			0.22
Ever smoker	22 (46%)	66 (60%)	
Never smoker	24 (50%)	42 (38%)	
Missing	2 (4%)	2 (2%)	
**Alcohol consumption**			0.029
Regular consumption	25 (52%)	81 (74%)	
No alcohol consumption	21 (44%)	26 (23%)	
Missing	2 (4%)	3 (3%)	

bioRxiv preprint doi: https://doi.org/10.1101/2025.11.23.690048; this version posted November 24, 2025. The copyright holder for this preprint (which was not certified by peer review) is the author/funder, who has granted bioRxiv a license to display the preprint in perpetuity. It is made available under aCC-BY 4.0 International license.

**Drinking hot beverages**			0.010
Yes	29 (60%)	88 (80%)	
No	14 (29%)	20 (18%)	
Missing	5 (11%)	2 (2%)	
**Diabetes**			1.0
Yes	1 (2%)	2 (2%)	
No	45 (94%)	106 (96%)	
Missing	2 (4%)	2 (2%)	
**HIV status**			0.052
Positive	17 (36%)	23 (21%)	
Negative	27 (56%)	64 (58%)	
Missing	4 (8%)	23 (21%)	

[1] P-values were calculated using two-sided Mann-Whitney U tests and Chi-squared tests as appropriate.

The following tables are included as a supplementary file:

Table S2 | Genus differential abundance unadjusted and adjusted beta-binomial regression results

Table S3 | ASV differential abundance unadjusted and adjusted beta-binomial regression results

Table S4 | ***Fusobacterium*** **ASV differential abundance unadjusted and adjusted beta-binomial regression results**
